# Design and characterization of Lactotransferrin peptide-loaded dextran-docosahexaenoic acid nanoparticles: an immune modulator for hepatic damage

**DOI:** 10.1038/s41598-023-40674-9

**Published:** 2023-08-19

**Authors:** Osama A. Madkhali, Sivakumar S. Moni, Muhammad H. Sultan, Mohammed Ali Bakkari, Yosif Almoshari, Emad Sayed Shaheen, Abdulrahman Alshammari

**Affiliations:** 1 Department of Pharmaceutics, College of Pharmacy, Jazan University, Jazan, Saudi Arabia; 2 Medical Research Centre, Jazan University, Jazan, Saudi Arabia; 3 Department of Pharmacology and Toxicology, College of Pharmacy, King Saud University, Riyadh, Saudi Arabia

**Keywords:** Drug discovery, Immunology, Gastroenterology, Nanoscience and technology

## Abstract

The primary objective of this research was to create injectable delivery formulations using Lactotransferrin (LTF) peptide-loaded dextran nanoparticles coated with docosahexaenoic acid. These nanoparticles, designated as LLDDNP, underwent a lyophilization process. The study encompassed a comprehensive investigation, including physicochemical characterization, in vivo assessment of biomarkers, and an examination of immune response through cytokine modulation. The zeta potential of LLDDNP was − 24.5 ± 12 mV, while their average particle size was 334.9 z.d.nm. The particles exhibited a conductivity of 2.10 mS/cm, while their mobility in the injectable dosage form was measured at − 3.65 µm cm/Vs. The scanning electron microscopy investigation, the lyophilization processes resulted in discrete particles forming particle aggregations. However, transmission electron microscopy analysis revealed that LLDDNP is spherical and smooth. The thermogram showed that about 95% of LLDDNP's weight was lost at 270 °C, indicating that the particles are extremely thermal stable. The XRD analysis of LLDDNP exhibited clear and distinctive peaks at *2θ* angles, specifically at 9.6°, 20.3°, 21.1°, 22°, 24.6°, 25.2°, 36°, and 44.08°, providing compelling evidence of the crystalline nature of the particles. According to proton NMR studies, the proton dimension fingerprint region of LLDDNP ranges from 1.00 to 1.03 ppm. The in vitro release of LTF from LLDDNP was found to follow zero-order kinetics, with a commendable R^2^ value of 0.942, indicating a consistent and predictable release pattern over time. The in vivo investigation revealed a significant impact of hepatotoxicity on the elevation of various cytokines, including IL-1β, IL-6, IL-8R, TNF-α, IL-2, IL-4, IL-10, and IFN-γ. Additionally, the presence of hepatotoxicity led to an increase in apoptosis markers, namely caspase 3 and caspase 9, as well as elevated levels of liver biomarkers such as CRP, ALP, ALT, and AST. In contrast, the treatment with LLDDNP modulated the levels of all biomarkers, including cytokines level in the treatment group extremely high significant at *p* < 0.001.

## Introduction

Liver failure is a progressive condition that develops over many years because of a variety of diseases, both infectious and non-infectious. The application of nanoparticles for the safe and effective delivery of medications to treat chronic liver diseases has rapidly emerged as a growing and promising area of research^1^. Lactotransferrin (LTF), a glycoprotein commonly present in mammalian milk, has garnered considerable interest as a promising therapeutic target. Notably, LTF exhibits the ability to bind with iron, playing a crucial role in the innate and adaptive immune functions^2^. The LTF peptide possesses a wide variety of properties, such as anti-inflammatory action that regulates the innate immune system, regulation of cellular proliferation, differentiation, and protection^3^. In addition, LTF demonstrates the pleiotropic mode action, which directly aids in the effect of macrophages for the expansion of T-helper cell divergence^4^. In contrast, the LTF is easily catabolized and has a short half-life. Antigen presentation cells must therefore be constantly stimulated and controlled to activate innate immune cells. LTF is endowed with multiple biological activities by recruiting antigen-presenting cells. Therefore, the LTF peptide nanoparticulate delivery system will be essential to target hepatic damage, rejuvenate the cells through the innate immune system, and induce CD4+ cells to develop adaptive immunity. In connection with the discussed problem, the nanoparticle drug delivery system is a promising tool to deliver drugs/vaccines/proteins in a safe and naïve form^5^. A recent review article indicates that nanosized immunomodulatory systems have been developed that allow immune cells to interact with infectious agents. This interaction enhances lymphatic drainage and endocytosis by overactive immune cells in infected areas^6^. Thus, nanomedicine is becoming increasingly important as a new treatment modality for various diseases^7,8^. In 2023, Fathil and Katas created silver nanoparticles that were simply complexed with LTF and DsiRNA before being packaged in gelatin hydrogels. The study showed that the pro-migratory, antibacterial, and anti-biofilm effects were superior^9^.

Dextran sulfate sodium (DSS), a natural polymer of α-1,6-D-glucopyranose, is hydrophilic, biodegradable, and biocompatible^10,11^. Dextran has also been demonstrated in nanomedicine, an innovative discipline that applies submicron particles for therapeutic and diagnostic purposes. The dextran sulfate has anti-coagulant, antiviral, and cholesterol-lowering characteristics^12–14^. In addition, dextran sulfate nanoparticles have an intense antibacterial action, as demonstrated in a previous study^15^. Docosahexaenoic acid (DHA), an omega-3 fatty acid, is utilized extensively in treating various human diseases. DHA has been linked to membrane-bound actions that improve membrane permeability by integrating with membrane phospholipids^16,17^. Recent research has shown that the nanovesicles containing recombinant HBsAg have strong immunomodulatory capabilities. he modulation of pro- and anti-inflammatory cytokine responses by HBsAg-entrapped DHA nanovesicles raises the possibility that recombinant HBsAg protein may be delivered through DHA nanovesicles^18^. Therefore, the present study focuses on the formulation of Dextran: DHA, a polymer lipid nanoparticle that will elicit a synergistic effect for delivering LTF peptide to prevent hepatic damage. Compared to the various studies previously published, the present investigation has yielded new and groundbreaking results. Our rigorous investigations and analyzes were previously undiscovered and have provided new insights into hepatoprotective agents. The novel formulation offers new perspectives, challenges existing theories, and paves the way for further research and development of hepatoprotection.

## Materials and methods

### Materials

In this study, we acquired DSS, MW (500,000) and sodium TPP, MW (367.86) from Santa Cruz Biotechnology, Inc., based in Dallas, USA. Furthermore, Docosahexaenoic acid was purchased from Sigma, located in St. Louis, MO, USA. For this research, all the necessary items were supplied by Ejadah Medical Supplies Est, located in Riyadh, Saudi Arabia.

### Formulation of nanoparticles

The nanoparticles were synthesized using a gelation technique and then coated with DHA. To produce these nanoparticles, a 1% w/v DSS was utilized as the polymeric carrier, along with a 1% w/v TPP as the stabilizer^13^. During the formation of nanoparticles, 0.5% w/v LTF human peptide was introduced at predetermined intervals. At 60 °C, the mixture was sonicated every 15 min and agitated with a magnetic stirrer for 60 min. Sonication was done at a predetermined time interval for 2 min at 100% amplification utilizing a laboratory probe sonicator, CPX ultrasonic processor (Cole Parmer Instruments Co, USA) during the mixing process. After the formulation LTF loaded dextran sulfate sodium nanoparticles were placed in 3 mL of docosahexaenoic acid for 60 min. Sonication was performed as described above at pre-determined time intervals. The mixture was placed in a beaker, kept on magnetic stirrer, and stirred for 30 min at 60 °C. Then the mixture was centrifuged at 2000 rpm for 20 min, and the DHA-coated nanoparticles (LDDNP) were settled down. The nanoparticles were collected by adding 0.25% v/v methanol in separate screw cap tubes and stored at 4 °C for further work. After an hour, the DHA-coated nanoparticles were filtered with Millex-GV Syringe Filter Unit, 0.2 m, PVDF, Merck KGaA, Darmstadt, Germany. Before being utilized in further studies, the filtrate was collected in a sterile glass screw-cap tube and refrigerated at 4 °C in a refrigerator.

### Physical and morphological characterization

The nanoparticles underwent lyophilization to enhance their stability for subsequent analysis. This lyophilized version of the nanoparticles was denoted as LLDDNP. To characterize the physical properties of these lyophilized LLDDNP nanoparticles in their injectable dosage form, crucial metrics such as zeta potential (ZP), particle size (nm; z.dnm), and polydispersity index (PDI) were employed. The physical characterization was conducted using the Nano-ZS Zetasizer from Malvern Instruments, UK. For filtration, PVDF membrane syringe filters with a pore size of 0.2 µm (Millex-GV Syringe Filter Un, Merck KGaA, Darmstadt, Germany) were utilized to filter the formulated nanoparticles. The morphological features of LLDDNP were examined using scanning electron microscopy (SEM) with a high-resolution Zesis scanning electron microscope. Additionally, transmission electron microscopy (TEM) with high resolution, using a JEOL transmission electron microscope, was also performed for further characterization.

#### Thermogravimetric Analysis (TGA)

The thermal stability of LLDDNP in an air atmosphere was investigated utilizing a thermogravimetric analyzer (TGA 8000, Perkin Elmer, Waltham, MA, USA). A sample of lyophilized DHA-DSS nanoparticles powder (10 mg) was carefully positioned on an aluminum pan for the analysis. The test was carried out by subjecting the sample to a gradual heating rate of 10 °C/min, over a temperature range of 50–300 °C.

#### X-ray diffraction (XRD) analysis

X-ray diffraction (XRD) analysis was employed to determine the crystalline structure of LLDDNP in the lyophilized DHA-DSS nanoparticles. The crystalline structure is an essential indicator of the nanoparticles' purity. The XRD analysis was performed using a Unisantis XMD 300 X-ray powder diffractometer (Unisantis Europe GmbH, Germany). During the analysis, XRD diffractograms were obtained in the *2θ* range of 2°–50°, using Cu K α radiation of incident beam (λ = 1.5418 Å) at a voltage of 45 kV and a current of 0.8 mA. A scanning range of *2θ/θ* was selected, and a scanning speed of 10 min^−1^ was applied to the powder sample. These XRD parameters allowed for the accurate examination of the crystalline structure and purity assessment of the lyophilized DHA-DSS nanoparticles.

#### Nuclear magnetic resonance (NMR) spectroscopy

The LLDDNP underwent NMR spectral analysis, and prior to the analysis, samples were prepared in deuterated water (D_2_O). The NMR spectrum of the crystal sample was recorded using a Bruker 400 Ultra shield NMR spectrometer, operating at 400 MHz for ^1^H-NMR and at 100 MHz for ^13^C NMR. Deuterated chloroform (DCCl_3_) was used as the solvent, and tetramethyl silane (TMS) served as the internal standard during the NMR analysis.

#### Loading studies

To prepare the LLDDNP for analysis, 1 g of the nanoparticles was added to 10 ml of an extraction medium comprising phosphate-buffered saline (PBS) at pH 7.4 and 10% of 0.1 N HCl. A small amount of tween 80 was added to the mixture, which was then placed on a hot plate with a magnetic bead and left at room temperature for 30 min. Subsequently, the mixture was subjected to centrifugation at 1000 rpm at 4 °C for 5 min. The resulting supernatant was collected for further analysis. For determining the loading of LLDDNP, enzyme immunoassay was performed using a LTF ELISA kit (DIA PRO, Milan, Italy). Based on the obtained data, the percentage of LTF loading (VL) was calculated using the following equation:

$$\% \;{\text{loading }} = \frac{{{\text{Total }}\;{\text{amount }}\;{\text{of}}\;{\text{ LTF}}\;{\text{ incorporated}}\;{\text{ in}}\;{\text{ LLDDNP}}}}{{{\text{Total }}\;{\text{weight }}\;{\text{of }}\;{\text{LLDDNP}}}} \times 100$$

### In vitro release profile

Exactly 100 mg of LLDDNP was carefully placed in a dialysis bag and introduced into 50 mL of phosphate-buffered saline with a pH of 7.4. To facilitate the dissolution process, a few drops of Tween 80 were added. The dialysis system was then positioned on a rotary shaker, running at 200 rpm. The entire experiment was conducted at a constant temperature of 37 °C, spanning a duration of 8 h. At regular intervals (0, 1, 2, 3, 4, 5, 6, 7, and 8 h), samples were collected from the dialysis bag and subjected to analysis using the LTF AB-IgG ELISA kit from MyBioSource, USA, which employs an enzyme immunoassay technique. By correlating the obtained optical density (OD) values against known LTF concentrations from a standard curve, we were able to determine the release profile of LLDDNP over the entire experimental period.

### In vivo animal study

This study utilized healthy male Wistar rats with an average weight of approximately 200 g. The rats were procured from the central animal facility of Jazan University, Jazan. Prior to the experiments, the animals were allowed to adapt to standard laboratory conditions, which included maintaining a relative humidity of 55–60% and a 12-h light–dark cycle. They had unrestricted access to pelleted feed and water during the acclimatization period. The rats were divided into four groups, with each group comprising six rats. The experimental protocol proceeded as follows:

- Group 1: Control group—In this group, the animals did not receive any treatment.
- Group 2: Product treatment group—The animals were intraperitoneally injected with 0.5 mL of the product (LLDDNP).
- Group 3: Sample control (Vehicle control)—The animals were intraperitoneally injected with 0.5 mL of Dextran- Docosahexaenoic acid nanoparticles (DDNP).
- Group 4: Standard control group—The animals were intraperitoneally injected with 0.5 mL of LTF standard.
- Group 5: Hepatotoxic group—The animals were induced with hepatotoxicity using CCl_4_.
- Group 6: Treatment group—After inducing hepatotoxicity, the animals were intraperitoneally injected with 0.5 mL of the product (LLDDNP) as a challenging method.

The immunogenicity study lasted for 16 days, following which blood samples were collected. The levels of cytokine network and apoptosis markers were determined from these blood samples. The blood was collected using a capillary tube from the retroorbital plexus, then transferred into sterile glass tubes and positioned at a slant to allow the serum to separate from the clotted blood. The serum was later extracted by centrifugation at 4000 rpm and stored at − 20 °C for biomarker estimation.

### Determination of biological markers

#### Serum IL-1β level

The concentration of IL-1β in the serum was assessed using a sandwich enzyme-linked immunosorbent assay (ELISA) with IL-1β rat ELISA kits (MyBiource, USA). The quantitative measurement of IL-1β in the serum was performed, and the endpoint was detected at 450 nm using an ELISA reader named ELx800 from the USA. To determine the IL-1β levels in the serum, the data were extrapolated based on a standard curve, and the results were expressed in picograms per milliliter (pg/mL).

#### Serum IL-6 level

The L-6 level was assessed by employing an IL-6 ELISA kit (MyBiource, USA), enabling the quantitative measurement of IL-6 protein in rats through a straightforward step-by-step ELISA test. The endpoint was swiftly identified at 450 nm using an ELISA reader called ELx800, manufactured in the USA. By extrapolating on the standard curve, the IL-6 concentration in the serum was determined and in picograms per milliliter (pg/mL).

#### Serum IL-8R level

The quantitative assessment of IL-8 R presence in the serum was conducted using a rat IL-8 R ELISA kit (MyBioSource, USA). This kit employs a sandwich enzyme immunoassay for the precise in vitro measurement of IL-8 R concentrations in rat serum. The endpoint of the assay was detected at 450 nm using an ELISA reader, specifically the ELx800 from the USA.To determine the level of IL-8 R in the serum, a standard curve was generated and used for extrapolation. The resulting concentration of IL-8 R was expressed in picograms per milliliter (pg/mL).

#### Serum TNF-α level

The rat serum TNF-α level was assessed by utilizing a rat TNF-α ELISA kit (MyBio-source, USA). This kit employs a sandwich enzyme immunoassay to detect and quantify TNF-α in rat serum in vitro. The endpoint measurement was obtained by reading the ELISA plate at 450 nm using an ELISA reader, specifically the ELx800 from the USA. The amount of TNF-α present was determined by extrapolating the data from the standard curve and subsequently expressed in picograms per milliliter (pg/mL).

#### Serum IL-2 level

The Quantitative assessment of IL-2 in serum was conducted using an IL-2 rat ELISA kit (ABCAM, USA). A sandwich ELISA approach was employed to determine the IL-2 levels in the serum samples. The quantification of IL-2 in the serum was measured at an endpoint of 450 nm using an ELISA reader, specifically the ELx800 from the USA. By extrapolating data from the standard curve, the IL-2 concentration in the serum was calculated and expressed in picograms per milliliter (pg/mL).

#### Serum IL-4 level

The quantitative analysis of serum IL-4 levels in vitro, a sandwich enzyme-linked immunosorbent assay (ELISA) was employed. The assay utilized a rat IL-4 kit from Abcam, USA. The endpoint measurement was obtained at 450 nm using an ELISA reader, specifically the ELx800 from the USA. The IL-4 concentration in the serum was calculated by extrapolating data from the standard curve, and the results were expressed in picograms per milliliter (pg/mL).

#### Serum IL-10 level

The level of IL-10 was determined quantitatively, a Rat IL-10 ELISA kit (Abcam, USA), was used. The ELISA technique employed a simple two-step process involving a capture antibody with an affinity tag and a detection antibody coated with a reporter. This immunocapture method allowed the measurement of IL-10 in serum samples. The quantitative measurement of IL-10 was obtained by detecting the endpoint at 450 nm using an ELISA reader, specifically the ELx800 from the USA. The IL-10 concentration in the serum was then calculated by extrapolating the data from the standard curve, and the results were expressed in picograms per milliliter (pg/mL).

#### Serum IFN—γ level

The in vitro ELISA for IFN-γ is a quantitative assay designed to measure IFN-γ protein levels in rat serum. The assay utilizes an affinity tag-labeled capture antibody and a reporter-conjugated detector antibody to specifically capture the IFN-γ analyte in the serum sample. The measurement is performed by detecting the endpoint signal at 450 nm using an ELISA reader called ELx800 (Bio Tek, USA). To determine the IFN-γ concentration in the serum, a standard curve is generated using known concentrations of IFN-γ. By comparing the endpoint signal of the unknown samples with the standard curve, the IFN-γ concentration in the serum is extrapolated and expressed in picograms per milliliter (pg/mL).

#### Serum apoptosis markers

Quantitative analysis of apoptosis markers, caspase-3 and caspase-9 was performed using the rat caspase-3 and caspase-9 ELISA kit (MyBioSource, USA). The ELISA kit utilizes a double-antibody sandwich enzyme immunoassay for accurate in vitro detection of caspase-3 and caspase-9 levels in rat serum. The endpoint measurements were taken at 450 nm using an ELISA reader, ELx800 (Bio Tek, USA). The levels of caspase-3 and caspase-9 were determined by extrapolating data from the standard curve and were expressed in nanograms per milliliter (ng/mL).

#### Assessment of serum markers for liver function

On the 16th day, the concentrations of CRP (C-reactive protein), ALT (Alanine Aminotransferase), AST (Aspartate Aminotransferase), and ALP (Alkaline Phosphatase) in the serum were measured using an automated clinical chemistry analyzer (Beckman Coulter, located in Brea, California, USA).

### Statistical analysis

The statistical analysis was performed using GraphPad Prism 9 software (Graph pad, USA), which is widely used for data analysis and visualization. Each reported value represents the mean of 6 batches (n = 6). The data is presented as mean ± standard deviation. The levels of statistical significance were considered as *p* < 0. 05, *p* < 0. 01, and *p* < 0.001, denoting the degree of significance of the results obtained in the study.

### Ethical statement

The present study underwent a thorough review process and obtained approval from the Institutional Research Review & Ethics Committee (IRREC) of the Faculty of Pharmacy, Jazan University, Jazan, Saudi Arabia. The approval number assigned to this study is 1108/152/1444. Throughout the research, strict adherence to the ARRIVE Guidelines for reporting in vivo experiments^19^ was followed, and all experiments conducted were in full compliance with the IRREC's guidelines.

## Results

### Physiochemical characterization

**Figure 1:**
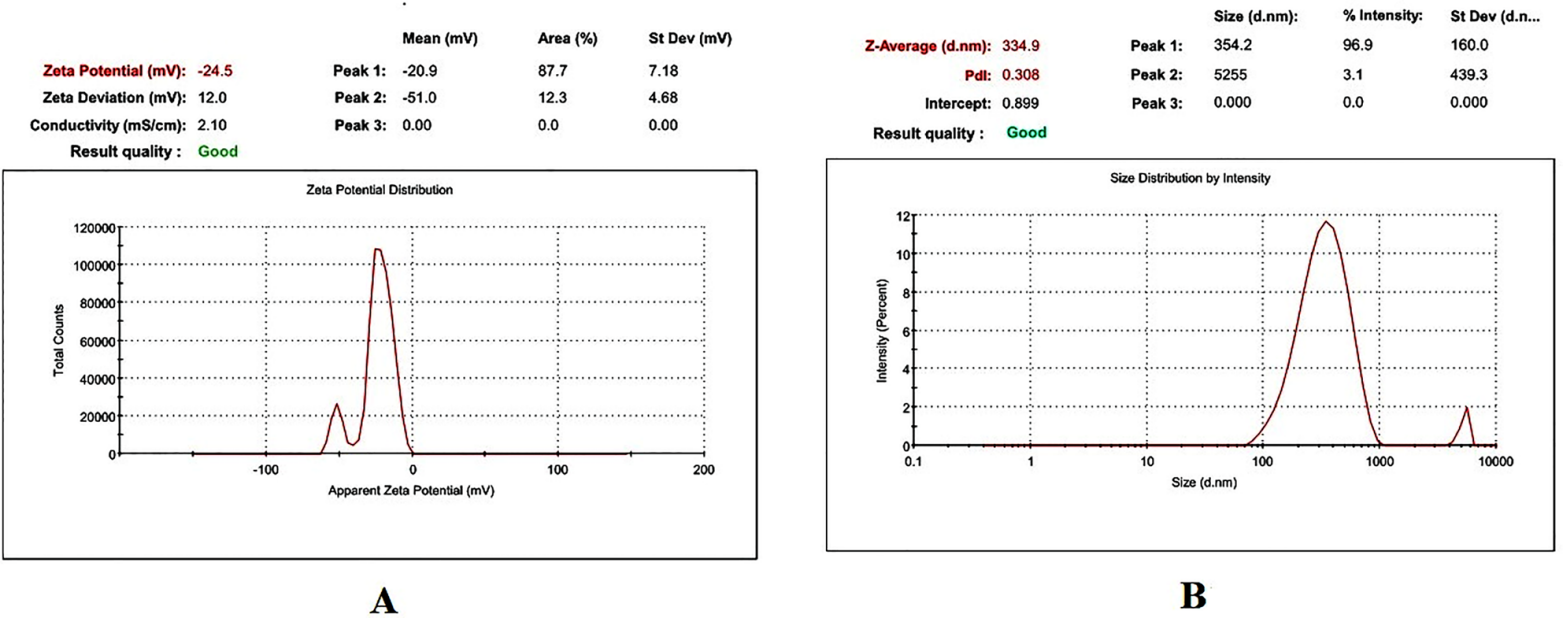
Physical characterization of lyophilized Lactotransferrin peptide-loaded dextran nanoparticles coated by docosahexaenoic acid (LLDDNP) (**A**) Zetapotential analysis of injectable LLDDNP (**B**) Size distribution analysis through intensity of injectable LLDDNP.

**Figure 2:**
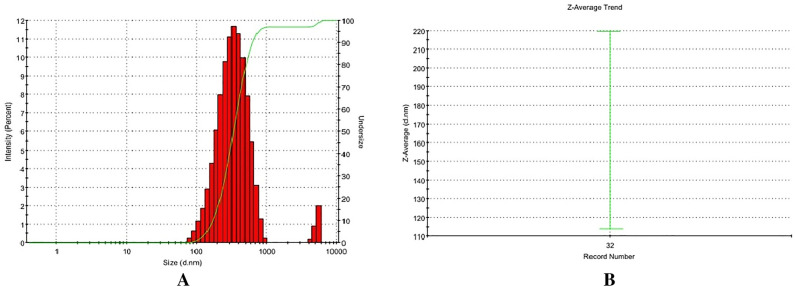
Physical characterization of lyophilized Lactotransferrin peptide-loaded dextran nanoparticles coated by docosahexaenoic acid (LLDDNP) (**A**) Size distribution analysis through percent intensity of injectable LLDDNP (**B**) Zeta average trend of LLDDNP.

**Figure 3:**
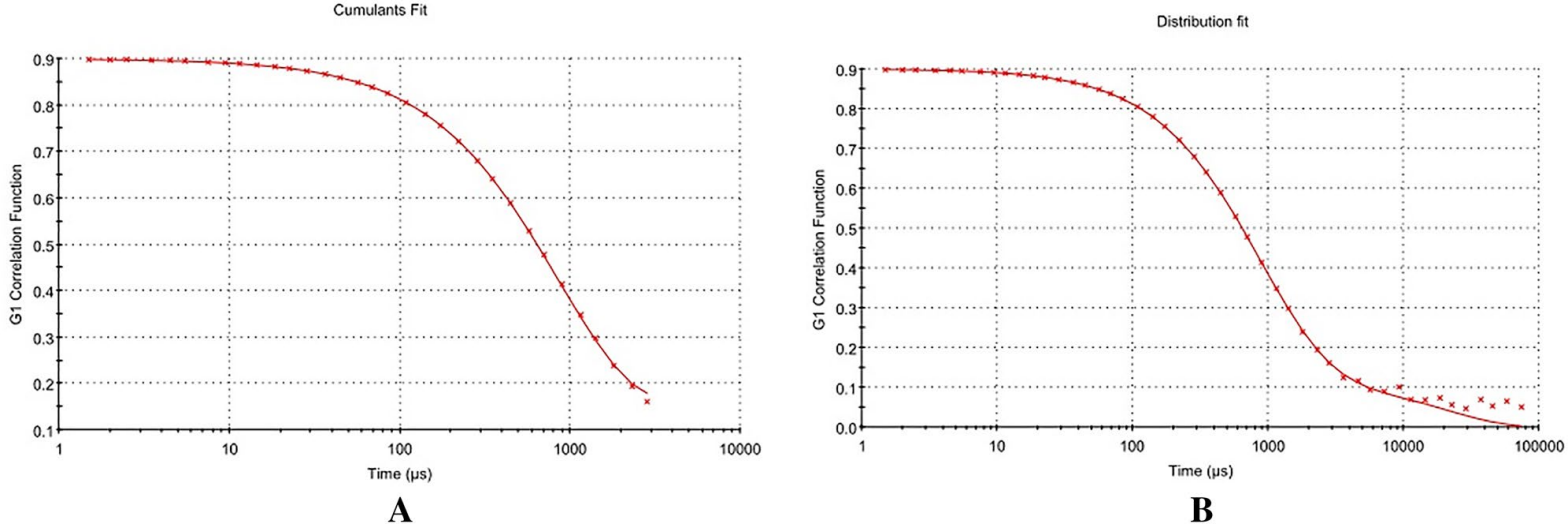
Physical characterization of lyophilized Lactotransferrin peptide-loaded dextran nanoparticles coated by docosahexaenoic acid (LLDDNP) (**A**) Cumulative fit analysis of injectable LLDDNP (**B**) Distribution fit analysis of injectable LLDDNP.

Table 1 presents the physicochemical properties of freely flowing LLDDNP, showcasing their potential through remarkable physical characteristics. Specifically, the injectable LLDDNP demonstrated highly favorable physical attributes in this research study. The physical characterization of LLDDNP through DLS analysis is representing in Figs. 1, 2 and 3. Peak 1 in Fig. 1, depicting the distribution of ZP of nanoparticles, showed that 87.7% of the particles were exhibiting − 20.9 ± 7.18 mV, and the remaining particles displayed − 51 ± 4.68 mV represented in peak 2 (Fig. 1A). The same two peaks could be seen in the size distribution through intensity (Fig. 1B). Interestingly, the average ZP value for LLDDNP was − 24.5 ± 12 mV (Table 1). The size distribution analysis represents a bimodal size distribution. The major peak at 354.2 ± 160 d.nm showed that the largest particles were 96.9% through the intensity distribution. The short peak displayed that the particle size was 5255 ± 439.3 d.nm, which showed the sedimentation forming during electrophoretic scanning. However, the zeta average of particles was observed as 334.9 z.d.nm. The PDI of injectable nanoparticles as colloidal formulation containing 1% w/v LLDDNP was determined to be 0.308, while the % PDI was 75.5, indicating that the formulation is homogenous. Figure 2A demonstrates various particle size distributions and serves as self-exemplary. Most particles had a size between 100 and 400 d. nm, whereas only a few particles had a size exceeding 1000 d. nm, as indicated by the size distribution's intensity peak. Figure 2B demonstrates the average zeta trend in nanoparticle size ranging from 110 d.nm to 220 d.nm. The cumulative fit analysis displayed an exceptional linearity (about 100%), and the size distribution fit reached approximately 95% in a colloidal dispersion system (Fig. 3A and B). The conductivity of the particle was determined as 2.10 mS/cm and the mobility of particles was showing − 3.65 µm cm/Vs demonstrating the particles low mobility in the colloidal system. However, the nanoparticles developed failed to form a crystalline structure but showed discrete particles (Table 1).

**Table 1:** DLS analysis of Lyophilized Lactotransferrin peptide-loaded dextran nanoparticles coated by docosahexaenoic acid.

Zeta potential (mV)	Particle size z.d.nm	% mass d.nm	Poly dispersity index (PDI)	% Poly dispersity	Y intercept value	Conductivity (mS/cm)	Mobility (µm cm/ Vs)
− 24.5 ± 12	334.9	19.23	0.308	75.5	0.899	2.10	− 3.65

**Figure 4:**
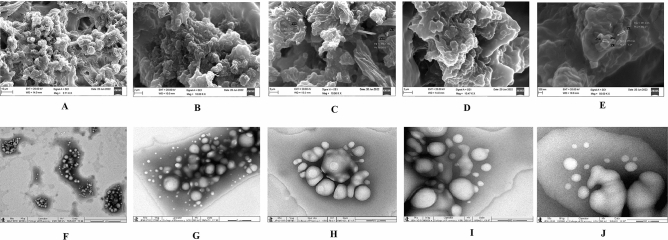
Morphological analysis of lyophilized Lactotransferrin peptide-loaded dextran nanoparticles coated by docosahexaenoic acid (LLDDNP) (**A**) Scanning electron micrograph of LLDDNP at × 2700 magnification. (**B**) Scanning electron micrograph of LLDDNP at × 10,000 magnification. (**C**) Scanning electron micrograph of LLDDNP at × 10,000 magnification. (**D**) Scanning electron micrograph of LLDDNP at × 10,470 magnification. (**E**) Scanning electron micrograph of LLDDNP at × 50,000 magnification. (**F**) Transmission electron microscope study of LLDDNP at × 6000 magnification. (**G**) Transmission electron microscope study of LLDDNP at × 12,000 magnification. (**H**) Transmission electron microscope study of LLDDNP at × 40,000 magnification. (**I**) Transmission electron microscope study of LLDDNP at × 60,000 magnification. (**J**) Transmission electron microscope study of LLDDNP at × 120,000 magnification.

The results of SEM and TEM analysis of LLDDNP are presented in Fig. 4. The LLDDNP is shown here at a magnification of 2700×, which reveals that the particles tend to have spherical shapes (Fig. 4A). The particles at a magnification of 10,000 showed few spherical-shaped and crystal-shaped nanoparticles (Fig. 4B–D). The nanoparticles were crystalline shaped with irregular morphology, and the size of particles was observed at 50, 000× magnification was 277.6–351.2 nm. In the present study, particle aggregation was observed after the lyophilization process in SEM analysis. In contrast, the particles as the nano formulation before lyophilization in the colloidal solution were analyzed TEM. The TEM study depicts a spherical shape and smooth surfaces at a magnification of 6000 × (Fig. 4F). The nanoparticles were smooth, spherical, and lucid distribution in the colloidal solution was non-homogenous in size under 12,000×, 40,000×, and 60,000× magnification (Fig. 4G). Non-homogenous spherical particle size was observed under 1, 20, 000 × magnification. A few particles were clubbed together and became unshaped. Both SEM and TEM studies showed highly porous particles.

**Figure 5:**
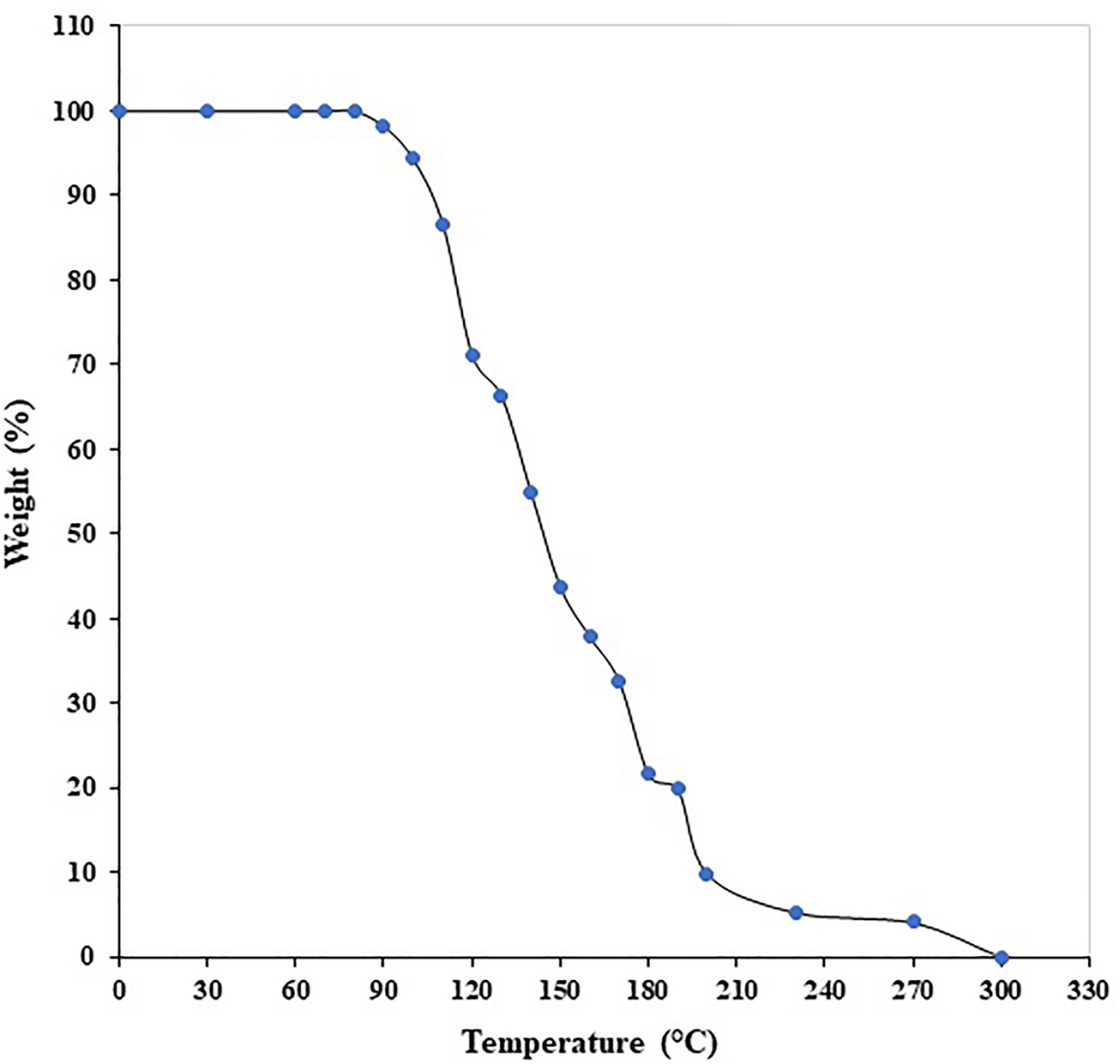
Thermogravimetry analysis of lyophilized Lactotransferrin peptide-loaded dextran nanoparticles coated by docosahexaenoic acid (LLDDNP).

**Figure 6:**
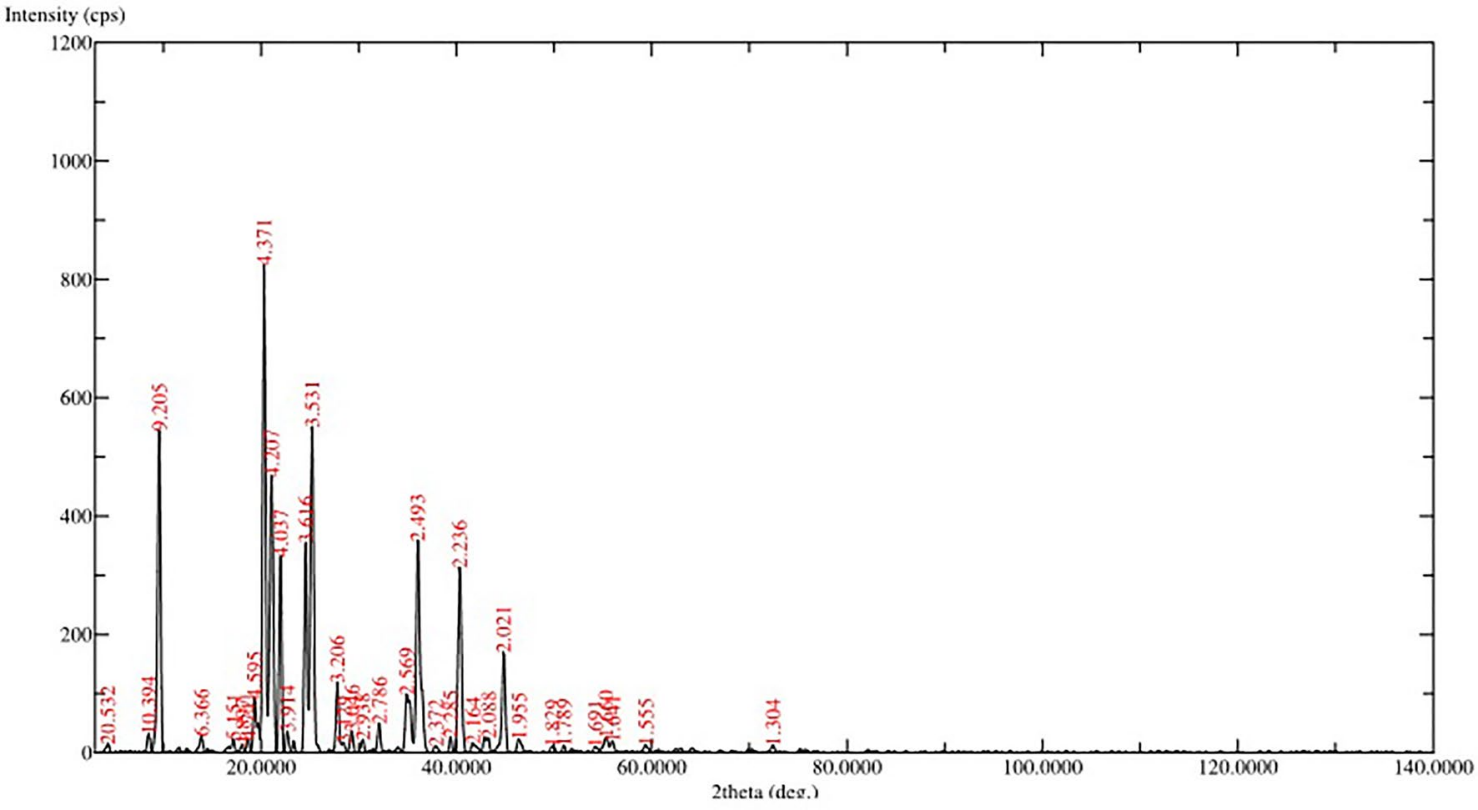
XRD analysis of lyophilized Lactotransferrin peptide-loaded dextran nanoparticles coated by docosahexaenoic acid (LLDDNP).

**Figure 7:**
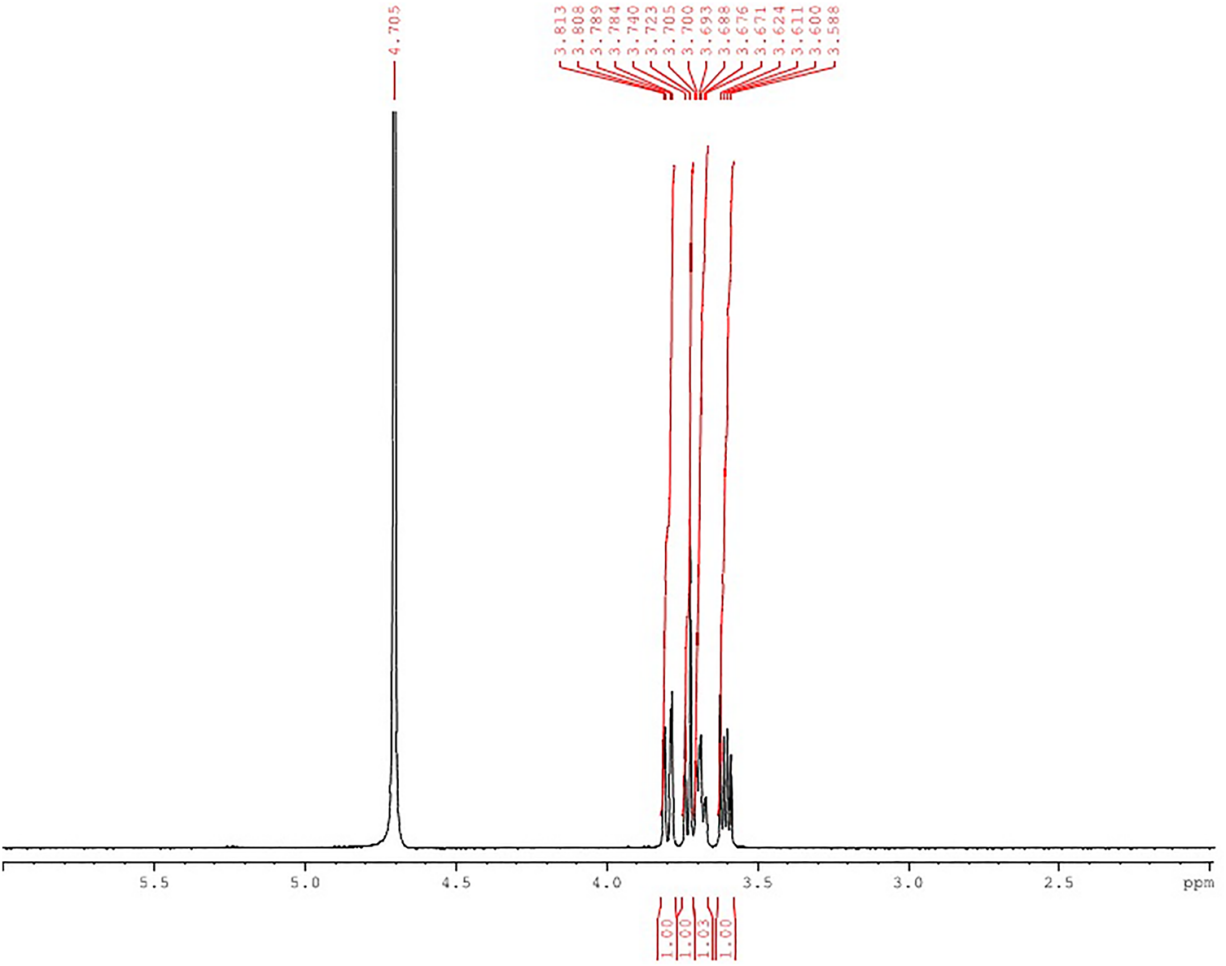
Proton NMR analysis of lyophilized Lactotransferrin peptide-loaded dextran nanoparticles coated by docosahexaenoic acid (LLDDNP).

TGA analysis was employed to assess the thermal stability of LLDDNP in the presence of oxygen. The results displayed a discernible peak, indicating a degrading effect. The thermogram indicated that LLDDNP experienced weight loss at a temperature of 270 °C, with approximately 95% of the weight being lost (Fig. 5). XRD measurements were utilized to examine the distinctive crystalline structures of nanoparticles. In the current investigation, the XRD analysis of LLDDNP at *2θ* clearly revealed individual nanoparticles through specific diffraction peaks. The diffraction peaks (d-values) observed were 9.205, 4.371, 4.207, 4.032, 3.616, 3.531, 2.493, 2.236, and 2.021. These corresponding d-values corresponded to *2θ* angles of 9.6°, 20.3°, 21.1°, 22°, 24.6°, 25.2°, 36°, and 44.08°, respectively. Moreover, the relative intensity (I/Io) values associated with these d-values were 67, 100, 57, 41, 44, 67, 44, and 21, respectively. The results confirmed the unique crystal lattice structure of LLDDNP**.** Interestingly, this study also showed many short peaks with d-values 2.569, 2.786, 3.206, 6.306, 10.394, and 20.532. The relative intensity of d values was 13, 7, 15, 4, 4, and 2, respectively. The corresponding 2*θ* for d values were 34.9º, 32.1º, 27.8º, 13.9º, 8.5º and 4.3º (Fig. 6). The ^1^H-NMR analysis gave the fingerprint region of LLDDNP from 1.00 to 1.03 ppm in the proton dimension. The most de-shielded proton peaks appeared at 3.58, 3.60, 3.61, 3.62, 3.71, 3.76, 3.68, 3.69, 3.70, 3.705, 3.723, 3.740, 3.784, 3.789, 3.808, 3.813 and 4. 705 ppm (Fig. 7).

### Loading and in vitro release profile

**Figure 8:**
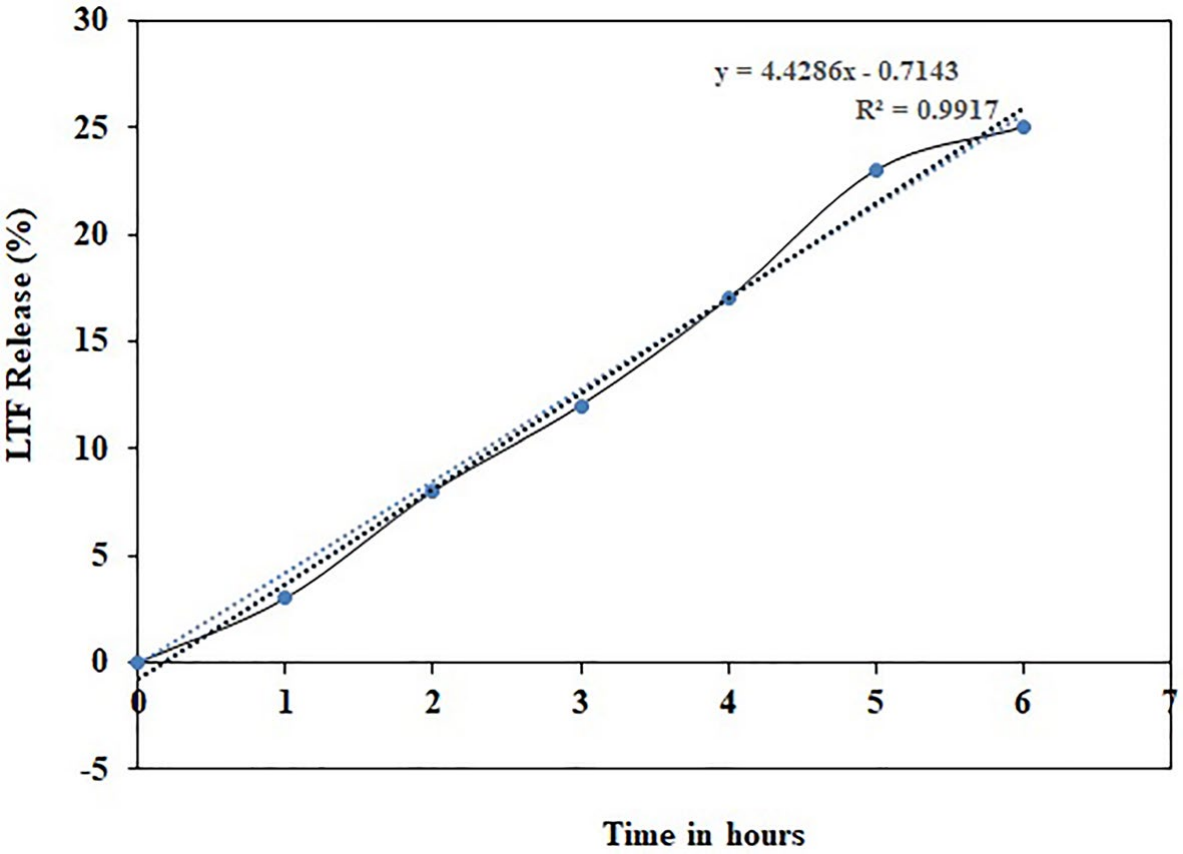
In vitro analysis of lyophilized Lactotransferrin peptide-loaded dextran nano-particles coated by docosahexaenoic acid (LLDDNP).

LTF was loaded effectively in LLDDNP with an efficiency of 98.0% ± 1.0%. Figure 8 displays the patterns of LTF peptide release from LLDDNP at pH 7.4. LTF from LLDDNP was released in a monophasic pattern. First-order kinetics characterized the release pattern, and the release profile's linearity was demonstrated by the r^2^ value of 0.94. LTF's release profile from LLDDNP was very consistent and sustained. In a 6 h experimental period, around 25% of LTF from LLDDNP was released into the releasing medium, demonstrating persistent release without any initial burst release.

### In vivo study

**Figure 9:**
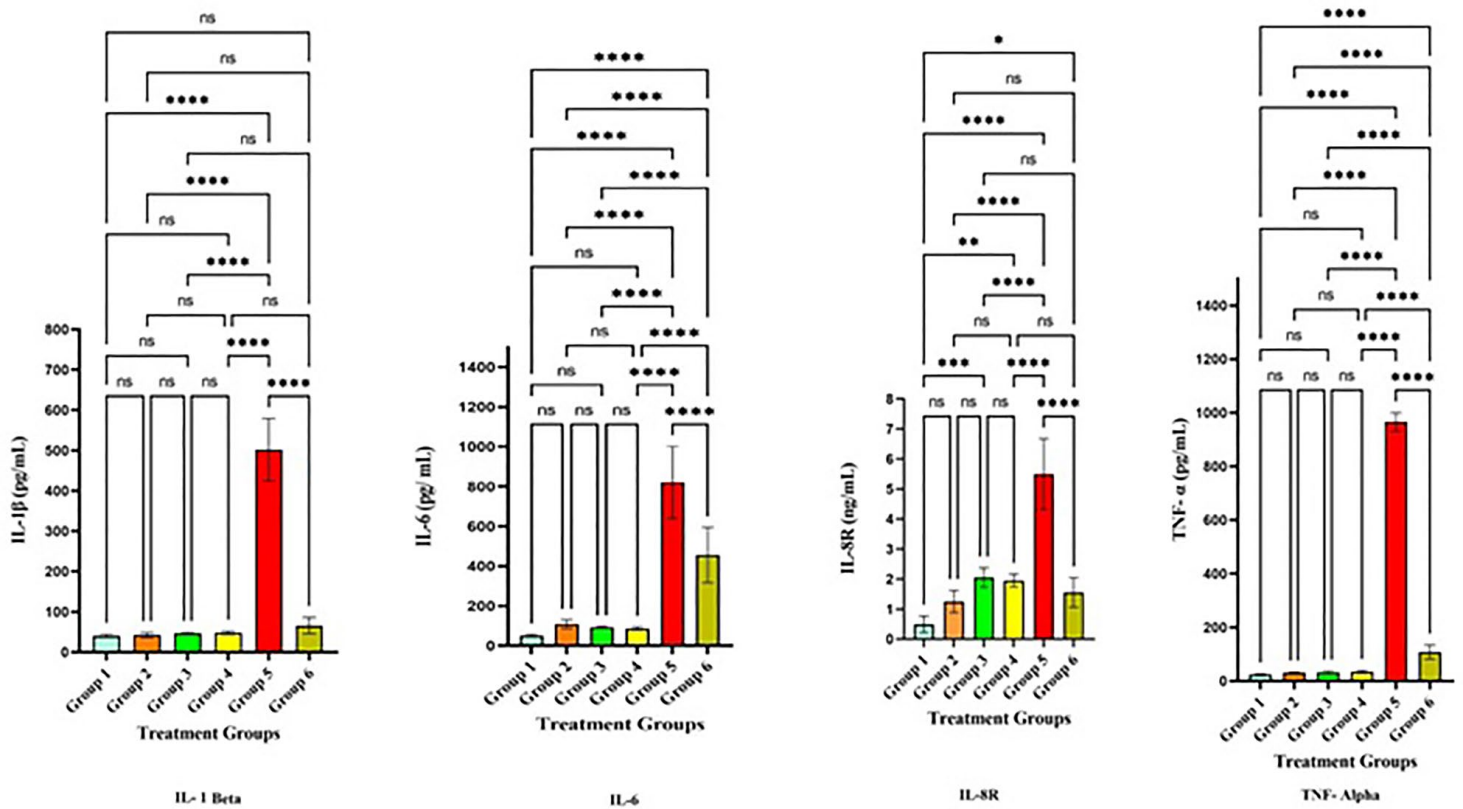
Proinflammatory cytokines level. **** Extremely high significant at *p* < 0.001, *** Highly significant at *p* < 0. 01, * Significant at *p* < 0.05, non- significant (ns) at *p* < 0.05.

Figure 9 provides a clear demonstration of the pro-inflammatory cytokine levels. Following the induction of hepatotoxicity, a considerable upsurge in IL-1β, IL-6, IL-8R, and TNF-α was observed in group 5 when compared to groups 1–4, with a significance level of *p* < 0.001. However, following treatment with LLDDNP (Table 2), a remarkable reduction in pro-inflammatory cytokine levels was observed in group 6 at a significance level of *p* < 0.001.

**Table 2:** Pro inflammatory cytokine levels.

Cytokines (pg/mL)	Group 1	Group 2	Group 3	Group 4	Group 5	Group 6
IL-1β	40.7 ± 4	42.5 ± 6.5	46.9 ± 2.9	48.3 ± 4.4	501.5 ± 77	65.9 ± 20
IL-6	51.4 ± 5.6	108.3 ± 21.5	93.8 ± 5.5	87.2 ± 8.9	821.2 ± 180.9	455.8 ± 139.6
IL-8R	0.4 ± 0.27	1.25 ± 0.3	2.1 ± 0.3	1.95 ± 0.21	5.5 ± 1.17	1.6 ± 0.5
TNF-α	23.71 ± 3.1	30.71 ± 3.6	32.71 ± 4.6	34.4 ± 5.8	965.9 ± 33.9	108.2 ± 26.7

Each value is the mean of 6 batches (n = 6) with a standard deviation.

**Figure 10:**
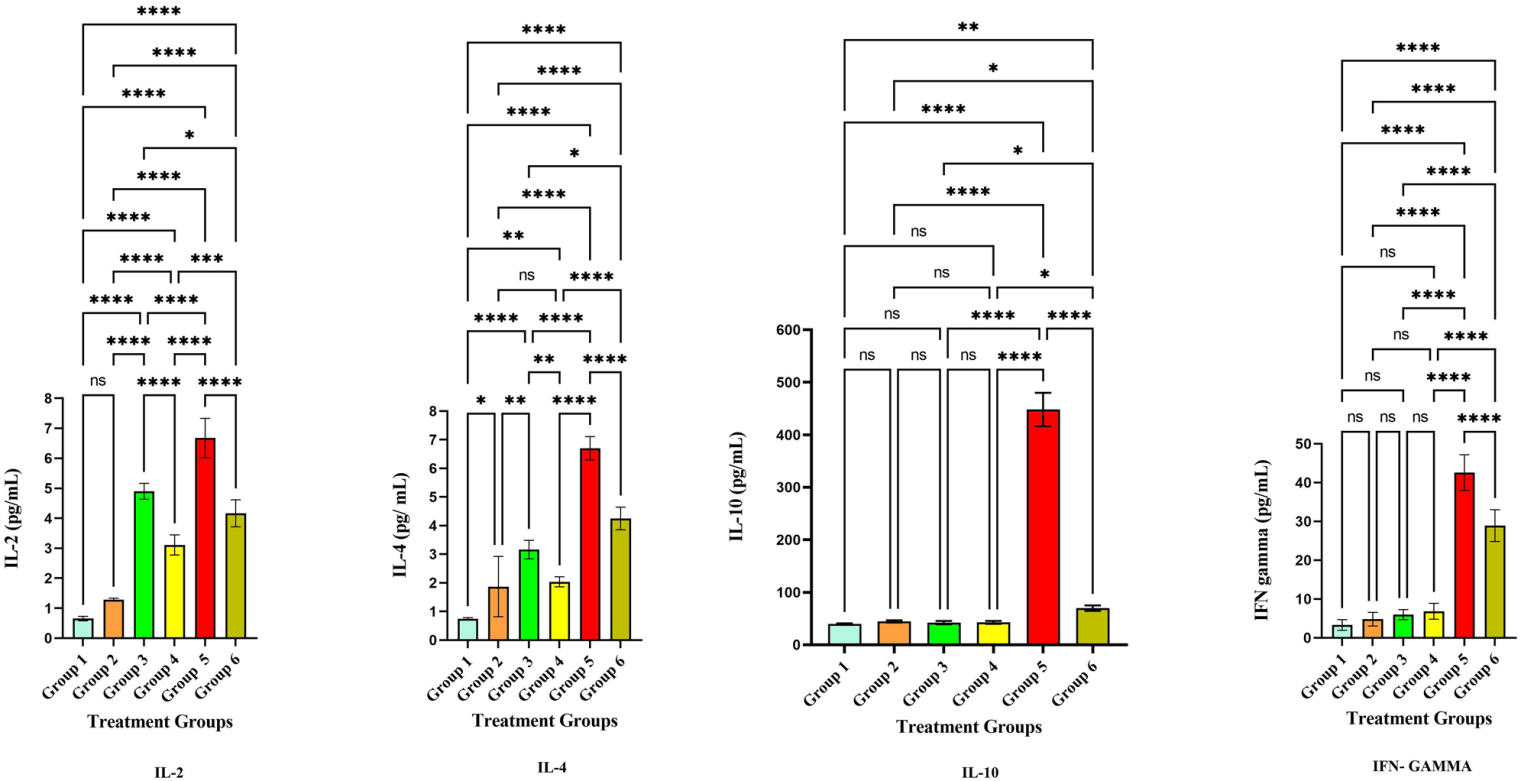
Anti-inflammatory cytokines level. **** Extremely high significant at *p* < 0.001, *** Highly significant at *p* < 0. 01, **Highly significant at *p* < 0. 05, * Significant at *p* < 0.05, non- significant (ns) at *p* < 0.05.

**Figure 11:**
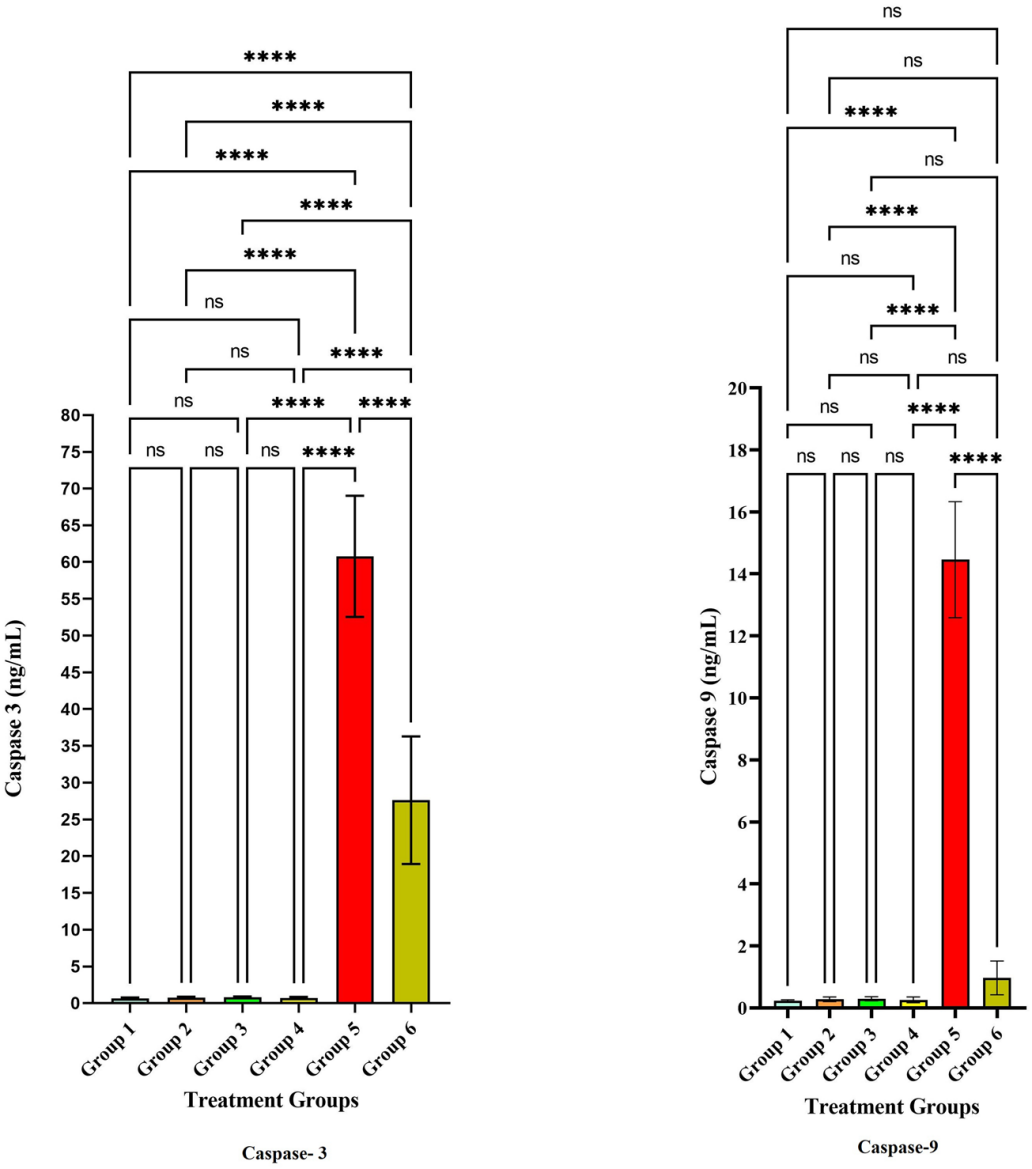
Apoptosis markers level**.** **** Extremely high significant at *p* < 0.001, non- significant (ns) at *p* < 0.05.

**Figure 12:**
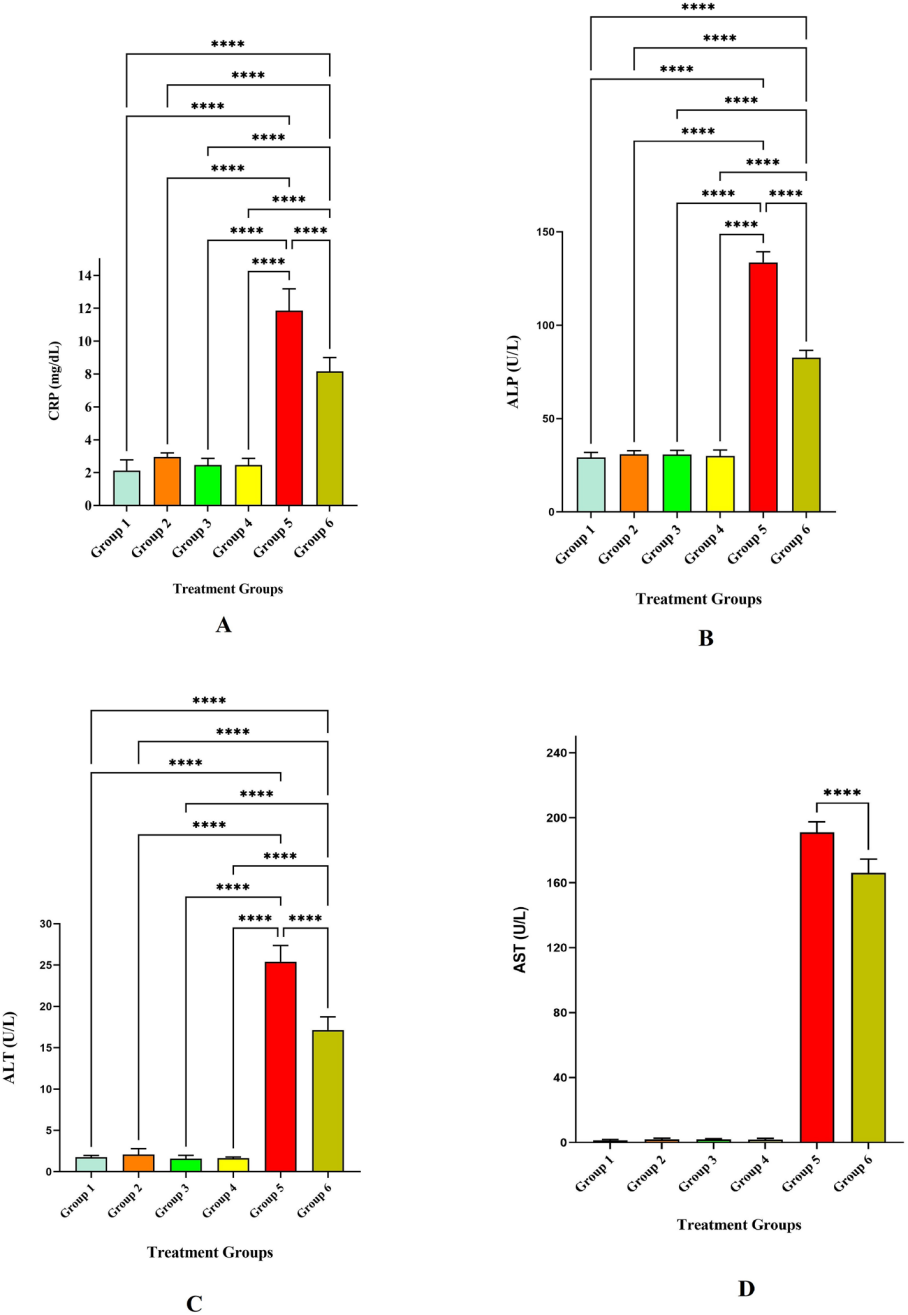
Liver Biomarkers level. (**A**) C-Reactive Protein (CRP) level (**B**) Alkaline Phosphatase (ALP) level (**C**) Alanine Transaminase (ALT) level (**D**) Aspartate aminotransferase (AST) level; **** Extremely high significant at *p* < 0.001, non- significant (ns) at *p* < 0.05.

The level of anti-inflammatory cytokines IL-2, IL-4, IL-10, and IFN-ℽ have significantly increased in the treatment groups. The level of anti-inflammatory cytokines is depicted in Table 3. The IL-2 level was significantly increased in groups 3, 4, 5, and 6 at *p* < 0.001 (Fig. 10). However, the level was modulated after the treatment of LLDDNP in group 6 and the level was reduced when compared to group 5 at *p* < 0.001 level (Extreme significant). The IL-2 level was significant at *p* < 0.05 on comparing group 6 and group 3. Interestingly, the level of IL-2 was less in treatment group 6 compared to the disease control group 5 at *p* < 0.001 level. The level of IL-4 level was also the same pattern followed like IL-2. On the other hand, the pattern of release of IL-10 was unique (Fig. 10). The IL-10 level was released very high compared to the rest of the groups. However, after treatment with LLDDNP the level of IL-10 was reduced in group 6 at *p* < 0.001 level. The IFN-ℽ was high in disease group 5 when compared to the rest of the groups. The level of IFN-ℽ was reduced significantly at *p* < 0.001 level. But the reduction of IFN-ℽ was less when compared to IL-10. Apoptosis markers caspases 3 and 9 were studied in this study, and the levels were presented in Table 4. The release of caspases 3 and 9 was extremely significant in disease group 5 at *p* < 0.001. However, the treatment with LLDDNP was significantly reduced in group 6 when compared to group 5 at *p* < 0.001 level (Fig. 11). It is interesting to note that when the levels of caspase 3 and caspase 9 were compared, the level of caspase 9 was significantly lowered when compared to the level of caspase 3 after being treated with LLDDNP in group 6. The results of research on the liver biomarkers CRP, ALP, ALT, and AST are presented in Table 5. Compared to the other groups, the amount of biomarkers was higher in group 5 (Fig. 12). The therapy with LLDDNP resulted in a considerable reduction, which was statistically significant at the *p* < 0.001 level. However, when compared to groups 1 to 4, the level of liver biomarkers in group 6 was much higher (Figs. 11 and 12); this suggests that the levels have not yet reached a normal level.

**Table 3:** Anti-inflammatory cytokine levels.

Cytokines (pg/mL)	Group 1	Group 2	Group 3	Group 4	Group 5	Group 6
IL-2	0.65 ± 0.07	1.28 ± 0.04	4.9 ± 0.3	3.11 ± 0.3	6.67 ± 0.6	4.16 ± 0.4
IL-4	0.75 ± 0.03	1.87 ± 1	3.2 ± 0.3	2.03 ± 0.2	6.7 ± 0.4	4.3 ± 0.4
IL-10	39.6 ± 1.3	44.4 ± 2.2	42.1 ± 3.2	42.8 ± 2.7	448.1 ± 31.9	69.8 ± 5.1
IFN-ℽ	3.3 ± 1.3	4.8 ± 1.7	5.9 ± 1.3	6.8 ± 2	42.6 ± 4.6	28.9 ± 4.1

Each value is the mean of 6 batches (n = 6) with a standard deviation.

**Table 4:** Apoptosis markers level.

Markers (ng/mL)	Group 1	Group 2	Group 3	Group 4	Group 5	Group 6
Caspase-3	0.67 ± 0.1	0.7 ± 0.1	0.8 ± 0.12	0.73 ± 0.12	60.8 ± 8.2	27.6 ± 8.6
Caspase-9	0.23 ± 0.03	0.28 ± 0.07	0.29 ± 0.06	0.26 ± 0.09	14.5 ± 1.8	0.96 ± 0.5

Each value is the mean of 6 batches (n = 6) with a standard deviation.

**Table 5:** Liver biomarkers level.

Markers	Group 1	Group 2	Group 3	Group 4	Group 5	Group 6
CRP (mg/dL)	2.12 ± 0.7	2.9 ± 0.2	2.5 ± 0.4	2.4 ± 0.4	11.8 ± 1.3	8.2 ± 0.8
ALP (U/L)	29.2 ± 2.7	30.8 ± 1.9	30.6 ± 2.3	30 ± 3.1	133.6 ± 5.7	82.5 ± 4
ALT (U/L)	1.75 ± 0.2	2.0 ± 0.4	1.6 ± 0.4	1.65 ± 0.12	25.4 ± 1.9	17.15 ± 1.6
AST (U/L)	1.3 ± 0.5	1.9 ± 0.6	2 ± 0.35	1.8 ± 0.7	191.06 ± 6.4	166.06 ± 8.4

Each value is the mean of 6 batches (n = 6) with a standard deviation.

## Discussion

The present study focuses on establishing the effect of LLDDNP on hepatoprotection in hepatoxic-induced Wistar rats. The average ZP of LLDDNP was − 24.5 ± 12 mV, indicating that the nanoparticle formulation was stable. Nanoparticles are highly stable colloidal suspension systems that inhibit nanoparticles from aggregating if they have zeta potentials that are either more than + 30 mV or less than − 30 mV. Our previous studies reported that the ZP of DSS nanoparticles exhibited as − 35.2 mV^15,16^. Furthermore, docosahexaenoic acid nanovesicles were exhibiting ZP of − 60.4 ± 10.4 mV^17,18^. Numerous studies on biodistribution have shown that smaller nanoparticles retain more than larger nanoparticles and that most nanoparticles collect in the Kupffer cells of the liver. The particle size ranged from 20 to 200 nm, circulating through veins and rapidly drained lymphatic drainage, providing better antigen presentation by resident dendritic cells^19–22^. Large nanoparticles of 500 to 1000 nm depended on tissue-resident macrophages and cellular transport by dendritic cells immigrating from the injection site. Therefore, LLDDNP developed in this study exhibited 334.9 z.d.nm indicating the ideal formulation for targeting immune cells. The PDI is a crucial metric that characterizes the spread of the particle size distribution in a colloidal solution. The injectable nanoparticle had a PDI of 0.3 and a PDI of 75.5%, indicating a monophasic nano injectable system. However, after formulation, only a few sediments developed, indicating that the particles had aggregated into larger groups, which was also reflected in the particle size distribution study. The value of the Y-intercept is an essential tool that can be used to evaluate the signal-to-noise ratio for measured samples. This ratio is expressed as a ratio between the signal and the noise. This ratio can be utilized as a metric for determining the accuracy of the data. In this experiment, the colloidal injectable dose form with a value of 0.899 for the Y intercepts was the most effective form overall. Usually, it is scaled so that an ideal signal will yield a value of 1, and a value greater than 0.9 is the best system, while the value of a good system should be greater than 0.6^23^.

The SEM studies demonstrated that the particles were clumped together and might be due to the lyophilization process since the outer layer of nanoparticles are made up of docosahexaenoic acid. Similarly, some studies suggested that the lyophilization process leads to particle aggregation^22–27^. A previous study showed that cryoprotectants had an impact on nanoparticle characterization, with mannitol having the greatest impact^28^. In the present study, mannitol was used as a cryoprotectant only for physicochemical characterization analysis. However, the final injectable nanoparticles were free from lyophilization.

Suleman et al.^27^ reported that silver- LTF (Ag-LTF) nanoparticles without caping with chitosan showed particle aggregation and capping with chitosan showed no aggregation^27^. Furthermore, the particles were highly porous and significant in releasing entrapped proteins. An earlier analysis revealed that using porous inorganic nanoparticles as medication carriers for cancer treatment could potentially improve life expectancy^30^. Mesoporous silica nanoparticles with extra-large holes may be a promising platform for developing cancer vaccinations^30^. A recent study revealed that at 259.78 °C, the lyophilized recombinant HBsAg-loaded DHA nanovesicles lost roughly 96.3% of their weight^19^. Previous research showed that the eicosapentaenoic (EPA)-rich oil was stable at temperatures up to 150 °C, with a 38% weight loss between 150 and 300 °C. Intriguingly, the study found that DHA-enriched oil had higher thermal stability than EPA-rich oil, with entire weight loss in the DHA-enriched oil occurring between 215 and 600 °C^31^. Therefore, TGA analysis demonstrated the thermal fragility of LLDDNP at high temperatures. Recent study demonstrated that the 2*θ* values of lyophilized recombinant HBsAg-loaded DHA nanovesicles were 9.9°, 20.6°, 25.5°, 36.3°, 38.1°, 40.6°, and 44.3°^18^ The multi-layered crystal lattice creation of nanoparticles was deduced from the distinctive pattern of LLDDNP's refractivity, which ranged from 2 to 9.2º. Decreases in the 2*θ* value of LLDDNP, on the other hand, suggested differing particle sizes in the colloidal injectable dosage form. These findings were reflected in the size distribution analysis as well. The *2θ* values of LLDDNP depict the crystalline particles. Further many min short peaks were observed in the diffractogram (Fig. 4), representing the influence of the lyophilization process after nanoparticle formulations. In a study conducted by Bakkari et al.^18^, it was found that fatty acids containing terminal methyl groups were detected in the NMR spectrum when the de-shielded proton peaks were observed at chemical shift values of 3.57, 3.58, 3.59, 3.60, 3.66, 3.67, 3.67, 3.67, 3.68, 3.70, 3.71, 3.77, 3.78, 3.79, and 4.71 ppm.

**Figure 13:**
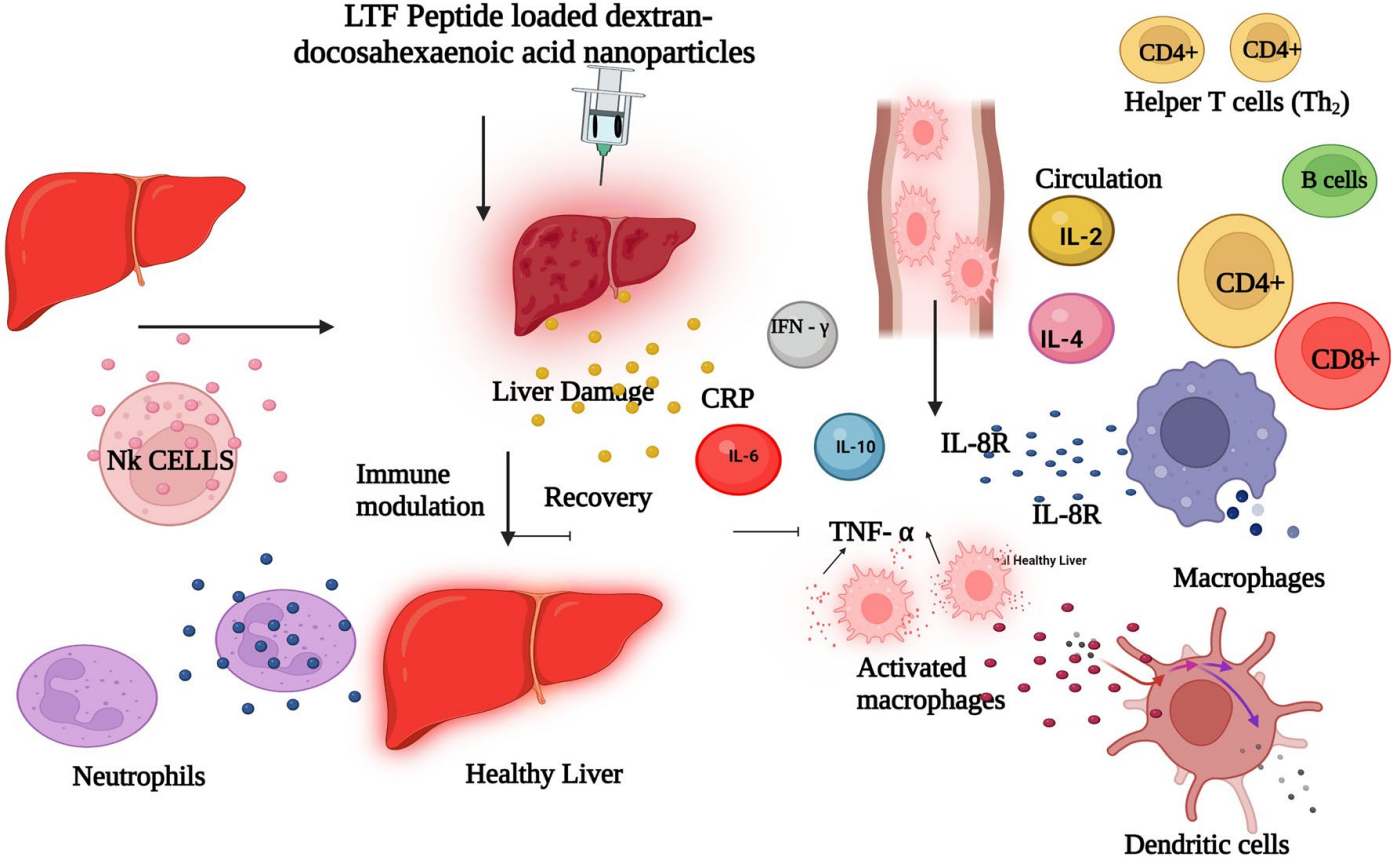
The mechanistic approach on the effect of lyophilized Lactotransferrin peptide-loaded dextran nanoparticles coated by docosahexaenoic acid (LLDDNP) through immune induction. This figure was created with BioRender.com, Bio Render, Canada.

The shift in the ^1^H-NMR spectra of the glycerol moiety of DHA from 3.58 to 4.71 ppm confirmed the presence of protons^32^. López-Machado et al.^33^ reported that lactoferrin released from nanoparticles exhibited sustained release and 83.6% after 48 h with r^2^ value was 0.99. In contrast, the current study demonstrates a faster release of LTF from LLDDNP. 25% of LTF was released for about 6h with excellent linearity fit showing zero-order kinetics. Figure 13 illustrates the mechanistic approach concerning the impact of LLDDNP, providing a clear and comprehensive understanding. The immune response is a highly intricate and regulated process that is triggered by the introduction of a protein antigen into the body. Historically, the liver was primarily considered a non-immunological organ, primarily involved in metabolic, nutritional storage, and detoxification processes. However, recent advancements have shed light on the fact that the healthy liver is also a site of complex immunological activity, orchestrated by a wide array of immune cells responsible for both pro-inflammatory and anti-inflammatory responses^34,35^. In cases of continuous liver injury, regardless of the underlying cause, there is a potential for the development of fibrosis, which can progress to severe conditions like cirrhosis and hepatocellular carcinoma. This process is influenced by several cytokines that play a crucial role in mediating hepatic inflammation, as well as the induction of apoptosis and necrosis in liver cells, cholestasis, and fibrosis resulting from this inflammatory response.

Interestingly, the same mediators are responsible for regenerating liver tissue after it's been injured. In this experiment, disease group 5 displayed highly elevated levels of the pro-inflammatory cytokines IL-1, IL-6, IL-8R, and TNF-α. However, treatment with LLDDNP led to a significant reduction and provided the foundation for a healing effect. Lactoferrin greatly reduced hepatic steatosis but did not affect visceral fat weight in a rat model of NASH. These results suggested that LF could protect against hepatic steatosis by regulating fatty acid metabolism in the liver or adipose tissue^36^.

On the other hand, anti-inflammatory cytokines such as IL-2, IL-4, IL-10, and IFN-ℽ also increased in the hepatotoxicity group (Group 5) but decreased after the treatment LLDDNP. An earlier investigation revealed that lower serum IL-4 concentrations accompany the acute and convalescent phases of hepatitis B virus infection. They claimed that the rise in IL-10 caused the IL-4 to level to fall. Furthermore, more significant T-lymphocyte activation may be possible during this time due to increased amounts of IL-2 and sIL-2R throughout the acute phase and until the full resolution of the HBV infection^37^. The drop in IL-4 suggested lower viral loads in the patients.

Additionally, because there was no discernible difference in the levels of IL10 between the patients and the controls, the condition was dormant. IFN- levels in patients significantly increased, which suggested that Th_1_ cells were activated^38^. In the present investigation, apoptotic markers caspase-3, and caspase-9 were elevated in the hepatotoxic treatment group (Group 5). Additionally, liver biomarkers, including CRP, ALP, ALT, and AST, were elevated following hepatotoxic induction. After treatment with LLDDNP, the elevated apoptosis and liver markers dropped dramatically, demonstrating potent immunomodulation. This may be related to the DHA coating of LLDDNP's dextran nanoparticles^39^. This study demonstrates that LLDDNP has a potent immunomodulatory effect and showed a promising hepatoprotective effect.

## Conclusion

Due to various factors, liver injury is one of the most prevalent problems of the current ultramodern lifestyle. The rise of liver biomarkers characterizes the pathological result of liver injury. However, the disease's molecular basis is intriguing due to increased pro-inflammatory cytokines and the caspase system. The current study suggests that LLDDNP effectively reduces hepatotoxic pro-inflammatory cytokines, apoptotic indicators, and liver biomarkers. Furthermore, through modulation of the cytokine network, LLDDNP plays a crucial role in liver cell rejuvenation. In addition, the physicochemical analysis of injectable LLDDNP demonstrated that it is suitable for therapeutic applications. Overall, the present study suggested that LLDDNP is a better therapeutic agent to combat hepatotoxicity and rejuvenate the liver possibly by modulating biomarkers.

## Data Availability

Data presented in this study is available from the corresponding author upon reasonable request.
